# Bleeding Bodies, Untrustworthy Bodies: A Social Constructionist Approach to Health and Wellbeing of Young People in Kenya

**DOI:** 10.3390/ijerph17207555

**Published:** 2020-10-17

**Authors:** Elizabeth Opiyo Onyango, Susan J. Elliott

**Affiliations:** 1 Department of Geography and Environmental Management, University of Waterloo, Waterloo, ON N2N 1N2, Canada; susan.elliott@uwaterloo.ca; 2 School of Nursing and Midwifery, Masinde Muliro University of Science and Technology, Kakamega 50100, Kenya

**Keywords:** wellbeing, health, young people, LMICs, Kenya

## Abstract

The Sustainable Development Goals provide a global development agenda that is meant to be inclusive of all people. However, the development needs for vulnerable populations such as youth are not reflected within the policy agenda of some developing countries. One of the reasons for this is that research that explores health and wellbeing concerns for young people are sparse in the region and where they exist, the focus has been on marginalized subgroups. To address this gap, this cross-sectional study explored the health and wellbeing of youth in Kenya. We conducted 10 focus group discussions and 14 in-depth interviews with youth ages 15 to 24 years. A thematic analysis of the data revealed that structural factors are important influencers of youth perceptions and their social constructions of health and wellbeing. Kenyan youth are concerned about the health status and healthcare services in their communities, as well as issues of community trust of youths and perceived risks of political misuse and emotional suffering. Our findings suggest that youth transitioning into adulthood in resource-constrained areas experience feelings of powerlessness and inability to take charge over their own life. This impacts how they perceive and socially construct health and wellbeing.

## 1. Introduction

In 2015, the United Nations (UN) adopted 17 Sustainable Development Goals that are meant to help track individual countries as they create blueprints for a dignified life, peaceful coexistence, and prosperity for their people now and into the future [1]. Countries are meant to work toward translating this shared vison into national development plans and strategies by 2030 [1]. While consultation processes have been an integral part in the development and identification of both the indicators for and the tracking of this new agenda, concerns remain around the representation of vulnerable segments of populations, including women, children, and youth [2].

The UN defines “youth” as persons between 15 and 24 years of age, and their inclusion or lack of inclusion in the development agenda directly impacts the health and wellbeing of both the current and the future populations of any country [3]. This is because these years encompass a critical phase in brain development in which lived experiences shape the capabilities of a person into adulthood [4]. During this stage of life, the foundations for physical, cognitive, social, emotional, and economic competencies are built, and these directly impact later life experiences [5] and the life trajectories of future generations. These capabilities are termed “triple divided” [6,7] and a significant length of impact suggests that a development agenda that improves the health and wellbeing of youth around the globe has the benefit of making the world a better place.

The adolescent life stage presents health and wellbeing challenges related to emotional control, behavioral and life choices, and the onset of sexual activity [5]. At the global level, youth face an increased commoditization of life, unemployment, instabilities at state and family levels, environmental degradation, and mass migration, all of which are associated with poor physical and mental health status [5,6,8]. The effects of these challenges are experienced differently in the high income and low-to-middle income countries [4], and the need for the development agenda to capture the contextual variations is imperative [8,9,10].

In the global North, a growing body of literature links the dominant western cultural values to the psychological wellbeing of young adults [8,10]. Twenge, Campbell [11], in a study of the changes in America’s children and adolescent’s mental health, showed that the modern western life characterized by individualism and competitiveness is causing more and more young people to become anxious and feel depressed. Furthermore, Furlong, Woodman [12] reveals that the changing economic and social conditions in the western countries where young adults are spending more time in post-secondary education and many are opting to delay marriage and starting of families have created a greater level of freedom and flexibility while promoting individualistic values. Characterized by preference for independence and continued engagement in the labor force, the individualistic lifestyle has led to weaker social relationships as younger adults become resistant to conform to the societal expectations of a collective lifestyle, thereby leading to inadequate health and wellbeing [13].

In the low-to-middle-income countries (LMICs) where over 240 million youth live [4], it is estimated that nearly one in every five young adults experiences poor wellbeing either in the form of physical and/or mental health disorders [9]. Experiences of early marriages deprive young people in LMICs of the ability to build their capabilities, with about 36% of girls aged 20 to 24 years marrying before the age of 18 years compared to 1% to 2% in high-income countries [14]. Over 30% (16 million) of girls aged 15 to 19 years in LMICs either in or outside marriage give birth every year [7]. Limited job opportunities, poor governance, and inadequate education systems which fail to equip young people with appropriate skills also negatively impact the health and wellbeing of youth in LMICs [15], causing mental health disorders among this vulnerable population [7]. These prevailing social and economic challenges, coupled with the penetration of the western cultural value of individualism, are posing new challenges in the world’s growing economies [5,6,8].

In Kenya, for example, sociocultural beliefs and practices, scarcity of resources, and poor governance limit opportunities to evaluate and address the health and wellbeing challenges of young people [16]. In these resource-constrained areas, healthcare service provisions and interventions tend to focus on infectious and childhood diseases, as well as emerging and rising non-communicable diseases [17]. As such, youth remain an overlooked group within health and social policy discussions in most LMICs despite the size of health challenges—both in terms of the number of persons affected and longevity of the impact [4]. Moreover, as young people in the developing countries move towards life goals focused on achievement of personal goals and become less concerned about each other within collective societies, new challenges to their wellbeing emerge. To address these new challenges, these countries need to collect more disaggregated data that are context-specific and that reflect the voices of youth as it relates to their health and wellbeing concerns [7]. The present study aimed to address this gap by exploring the health and wellbeing indicators that matter to young people in Kenya.

While there have been previous attempts to understand the health and wellbeing issues among Kenyan adolescents, the existing studies have focused on specific aspects of adolescent health such as reproductive health [18], menstrual hygiene [19], and sanitation [20]. Some have focused on specific vulnerable groups such as pregnant adolescents [21,22] and youth living with HIV [23]. Limited studies that focus on more normative environments like the general youth population and to how they socially construct health and wellbeing exists in Kenya. With the changing sociocultural systems such as the education system and growing tribalism as facets of Kenyan societies, young people are experiencing poor psychosocial health and wellbeing.

Kenya’s education system is founded on competition and achievement of better grades to increase prospects for a better life. Young adults are therefore experiencing increased psychosocial distress due academic pressure and ethnocentric politics [24]. Although Kenya is an ethnically diverse country, with at least 42 tribes, over the past six decades, major tribes (e.g., the Kikuyu, Kalenji, Luo, Luhya, and Kamba communities) have used tribal identities and affiliation to consolidate political and economic power, thereby secluding the minor tribes [25]. The ethnocentric governance has led to distrust and tribalism which has left the majority of the Kenyan population struggling to gain meaningful education, employment, infrastructural development, and other resources. In some instances, ethnic politics have led to tribal clashes where young people have been used by the political elites to gain political mileage and to cause instability [26,27]. It is possible that discrimination due to ethnicity and the disintegration of the collective nature of the Kenyan societies is significantly impacting young people’s experiences and perceptions on health and wellbeing [16].

With the 2003 free primary education policy and subsidized secondary education, the proportion of young Kenyans attending secondary school has risen sharply, and many are spending more time in school with the hope of higher-paying jobs and better life prospects [28]. At the same time, as the world experiences environmental changes and agriculture becomes more automated, engagement in farming is not a viable employment option for the youth [29]. However, with limited employment opportunities, adolescents in Kenya are consistently frustrated, thus depicting poor psychosocial health and wellbeing outcomes. As Kenya moves towards achievement of the UN global development agenda, initiatives aiming at understanding how youth socially construct their health and wellbeing amidst the several sociocultural and environmental changes are imperative [30]. To address this knowledge gap, we employed Krieger’s ecosocial framework to explore meaning and perceptions of health and wellbeing of young Kenyans.

### Ecosocial Model in Exploration of Health and Wellbeing of Youth in Kenya

**Figure 1:**
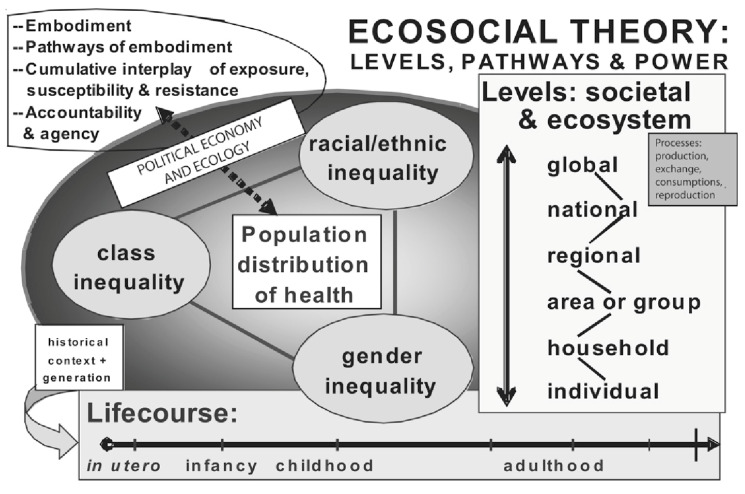
A heuristic diagram for guiding ecosocial analyses of disease distribution, population health, and health inequalities—a model for exploring health and wellbeing, reproduced from Krieger [31] with permission from the American Public Health Association.

The ecosocial theory by Nancy Krieger [31] informed the overarching conceptualization of this research. Krieger’s theory is founded on the poststructuralist perspectives which allowed for a critical analysis of who and what drives overall patterns of health and wellbeing (Figure 1). As demonstrated in studies of marginalized groups [32,33,34], the ecosocial model suggests that people literally embody their lived experiences in societal context throughout their life course and that there are multiple interconnected pathways within and across societies such as patriarchy, capitalism, social class, and other societal arrangements of power and social systems. The social structures determine the extent to which different societal groups have access to functional and basic amenities and resources including employment, sanitation and hygiene services, medical care, social networks, and education [31]. Consequently, the systemic and individual characteristics determines people’s susceptibility and experiences of health and wellbeing.

Adolescents in Kenya, for example, conceptualize health and wellbeing around issues of economic opportunities, state of the environment and neighborhoods, political engagement and stability, healthcare services, and social interactions—including trust and emotional wellbeing. With the experiences of historical and environmental injustices evident in political violence and high unemployment rates, the male youth bodies are associated with crime and the youth feel the lack of trust in the community. The female youth, on the other hand, are delicate bodies at risk of sexual violence; thus, they need to be regulated and protected. Additionally, with the changing social and economic structures, young Kenyans, especially those in the formal settlements, experience the weakening of the collective societal systems as parents spend more time in economic activities. The current generation are therefore growing with limited parental and societal support. However, the framing of health and wellbeing vary regionally and by social class and ethnicity as youth from tribes that have never ascended to power feel that they are left out because of their tribal–political inclination.

Employing the ecosocial model in a study of health and wellbeing of young adults provides a unique contribution to the knowledge on how youth conceptualize and perceive health and wellbeing. This is important because previous studies have mainly focused on specific health issues and the associated individual level factors that affect some marginalized youth such as those living with HIV, teenage mothers, and institutionalized youth. The current study explores young people’s perception and meaning of health and wellbeing as it relates to both the structural and the individual level factors in the broader population. This is important as it exposes the other factors important to the youth in defining their health and wellbeing and that are essential in policy formulation and evaluation.

## 2. Materials and Methods

### 2.1. Research Design

This was a qualitative study that drew from social constructionist and interpretive philosophies, which place emphasis on the subjective accounts of participants’ lived experiences [35], the researcher’s interpretation of participants’ narratives, and a critical analysis of such narratives [36]. This research orientation also ensure that meaningful results are generated through interviewer–interviewee interactions [37]. The purpose of the study was to gain a deeper understanding of the health and wellbeing of young people and to document perceptions and meanings of healthy community and a good life. Participants were specifically asked questions about what makes for a healthy community and a good life and that if a new community was to be started, what things would they want to be put in place to ensure a healthy community and a good life? The questions were based on John Eyles’ health and wellbeing mapping tools for the determinants of health among the First Nations of Canada [38,39].

### 2.2. Selection of Study Site

**Figure 2:**
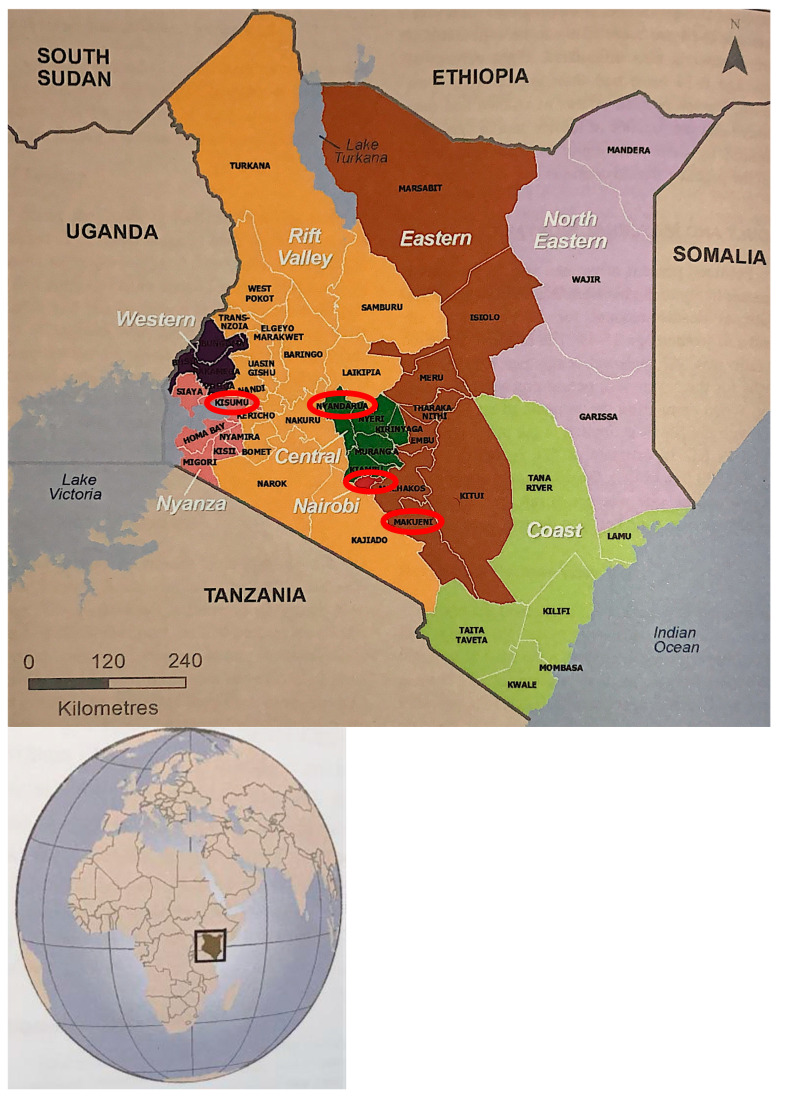
The map of Kenya showing provincial and county boundaries. Note: Red rings show specific counties where the research was conducted.

The study was conducted in Kenya between January and April 2017. Kenya is a LMIC located in on the eastern side of sub-Saharan Africa (Figure 2). Kenya is governed by a federal government system with 47 counties (Figure 2). The population of Kenya is young, with approximately two in every ten (19%) Kenyans aged 15 to 24 years [40,41]. Many Kenyans (40%) live below $1.25 a day, and as a result, many experience social and health inequalities in relation to accessing basic amenities such as water, sanitation facilities, food, and decent housing [14]. While the urban residents, especially those in the formal settlements report better wealth index and health outcomes, populations in arid to semi-arid areas and poor rural settings are worse off in terms of their wealth quintile and overall wellbeing.

To capture these diversities, our research was undertaken in four counties: Kisumu, Nairobi, Nyandarua, and Makueni. The counties were purposively selected based on a constraint of urban areas, with a diversity of socioeconomic status, and arid and semi-arid conditions. Kisumu County in Nyanza Province is mainly inhabited by the Luo; it is a relatively poor region that has consistently been in opposition government since Kenya’s independence. The region has a low housing quality index (7.9%), low access to improved water and sanitation services (38%), and electricity and household asset ownership below the national level. Nyandarua County, on the other hand, is in Central Province and is a relatively wealthy region with a housing quality index (38%) second only to Nairobi County [42]. Nyandarua County also has access to piped water and improved sanitation services, access to electricity, and household asset ownership above the national level. To represent the arid to semi-arid regions, we selected Makueni County in Eastern province. Because of the climatic conditions, Makueni county is relatively poor with at least four in every ten households reporting a lack of food (or money to buy food) and clean drinking water [42]. Nairobi County in Nairobi Province was selected to represent an urban area with both formal (i.e., Langata gated community) and informal (i.e., Kibera slums) settlements.

### 2.3. Selection of Participant Recruitment

Study participants included youth as defined by the UN (males and females aged 15 to 24 years) [43]. Purposive sampling was used in the selection of young people who were invited to an information session. The information sessions were organized by gender to allow for homogeneity within and heterogeneity between the groups. Individuals who had lived in the area for not less than five years and who gave informed consent were included in the study. The presumption was that such individuals held a depth of experiences of health and wellbeing within their communities, which would allow for an in-depth exploration of health and wellbeing indicators that matter to the youth. In the Langata area, a formal settlement in Nairobi County, enough numbers for the male youth to participate in FGD was not achieved and this was compensated for by in-depth interview (IDIs) with male youth representatives. For the IDI participants, a similar recruitment process was used, and information sessions were conducted with individuals who were interested in participating after receiving the invitation letter and having a telephone conversation with a researcher.

### 2.4. Data Collection Methods

Focus group discussion (FGD) and semi-structured in-depth interview (IDI) were used as methods of data collection. To ensure quality data collection and to allow the researcher to gain confidence and familiarity with the conduct of FGDs, an experienced moderator was hired for the first two FGDs where the researcher remained a notetaker and a co-moderator. In the remaining FGD, a notetaker was hired and one of the researchers was the moderator. The moderator was responsible for guiding the discussion while the notetaker was responsible for noting down both verbal and nonverbal responses and ensuring that the tape recording was ongoing. The interviews took place in the community either in a church, a hall, or an institution of learning. Although all the interviews were conducted either in Swahili and/or English, the two national languages in Kenya, Luo—a local dialect in the Nyanza province—was a preferred language by the participants in Kisumu County. To ensure accurate translation and to minimize loss of information through language translations, the corresponding author who is fluent in all the three languages immersed herself into the data from collection, transcription to analysis, and revisited the audio-recorded interviews whenever required. Each FGD lasted approximately 90–120 min and consisted of between 8–12 participants. The FGDs were held separately for male and female youth to allow for free expression of view during the discussion [44]. The participants were asked to treat the discussions as confidential, but since this could not be guaranteed in the FGD, the researcher asked participants to disclose only those things that they would feel comfortable being repeated outside the group [44]. In-depth interviews were conducted by a single researcher and took about 40–60 min. The researches also made sure that IDIs were audio recorded and took short notes as the interview progressed. Both FGDs and IDIs explored questions about what makes for a healthy community and what matters most in having a good life.

### 2.5. Data Management and Analysis

The audio-recorded interviews were transcribed verbatim by the correspondent author (one of the researchers) and, if necessary, translated from either Luo or Swahili into English. Initially, the plan was to have a translator in the interviews, but it became apparent that the translation was not verbatim and that having a translator was costly. Furthermore, one of the researchers was well conversant with all the languages used in the interviews and it made more sense to have the researcher transcribe the audio records. The transcripts were typed out and each transcript was saved as an individual word document with clear labeling showing the study site, type of interview, and respondents’ sex. The transcripts were saved on a password-protected computer only accessible to the research team.

Using both inductive and deductive methods, we developed a thematic code set, allowing for predefined and new themes to emerge [45,46]. The predefined themes were based on ecosocial theory pathways of embodiment, which suggest that socio-structural factors interact with an individual’s biology to create actual and perceived health outcomes. The theme codes, together with the transcripts, field notes, and memos, were then assembled and organized in NVivo (QSR International, version 11.2, Melbourne, Australia) and the data coded line by line. Matrix coding and cross tabulation analysis were used to explore the data to identify unique social constructs by gender and region. To validate the coding and analysis of the data, an independent researcher (a peer researcher) cross-checked the analysis for accuracy and consistency. The process of peer checking created opportunities for multiple interpretations and understandings and for broader connections within the data [44]. To ensure trustworthiness, the data were triangulated in three main areas: (a) we collected data from different data sources; (b) we used different data collection methods (IDIs and FGDs); and (c) we used different theoretical models—ecosocial and the social constructionist lens—to inform the research process.

### 2.6. Ethical Considerations

This study commenced after receiving ethical approval from the University of Waterloo Ethics Review Board (ORE no.: 21946) and the Masinde Muliro University of Science and Technology Ethics board (MMU/COR: 403009 [56)) in Kenya. Local administrative authorities served as community entry points. Area chiefs were asked for permission to conduct the research within their jurisdiction and they suggested community gatekeepers who were mostly community health volunteers to assist with participant recruitment. Potential participants were individually contacted through invitation letters to an information session. Invitation letters were accompanied with an information sheet detailing the research process, the research objectives, expectations, and the merits and demerits of participating. A day before the scheduled information sessions, a researcher made phone calls to the potential participants to remind them of the meeting and to invite others who might be interested in the study. After a detailed information session, potential participants gave an informed consent. Each participant also received an information sheet with the researcher’s contact. Each participant agreed to written and verbal consent. The respondents were assured of privacy and confidentiality and that the collected data would only be accessible to the research team. Throughout the research process, voluntary participation was emphasized, and participants were informed of their right to refuse to participate or to respond to any question or withdraw from the discussion without any consequences.

## 3. Results

### 3.1. Perceptions, Meanings, and Social Construction of a Healthy Community and Good Life

In this section, we present the findings of how youth in Kenya perceive, define, and socially construct the health and wellbeing of their communities. A total of 10 FGDs were held with young people aged between 15–24 years, five with girls and five with boys. With regards to the IDIs, we held a total of 14 interviews with young people, out of whom six were girls and eight were boys, aged 15–24 years. (See Table 1 for participant characteristics.) For the IDIs in Langata, a formal settlement in Nairobi, we interviewed more boys than the girls to compensate for the challenge of recruitment of young boys for the FGD. In Nyanza province, we conducted more female IDIs not only for similar reasons but also for saturation of data to be achieved. The number of interviews in the other regions were also determined by saturation—that is, until when no new information was emerging.

The findings reveal that perception and meaning of a healthy community and wellbeing, or “a good life”, that young Kenyans adopt are context-specific and are informed by prevailing socioeconomic, historical, political, and environmental conditions that directly or indirectly affect their wellbeing and associated health outcomes. The two concepts, a healthy community and wellbeing (a good life), were, however, used interchangeably and defined using similar concepts. Participants in this study defined a healthy community and having a good life in relation to the systemic factors that either promote or limit opportunities and freedoms available and the associated impacts on health and wellbeing. Issues of economic opportunities, state of the environment and neighborhoods, leadership and governance, social interactions, and relationships, as well as community health status and quality of healthcare services, were considered important in having a healthy community and a good life. Given the current prevailing socioeconomic and cultural changes, the young people adopted specific concepts and themes to describe their bodies. They referred to their bodies as “bleeding bodies”, “untrustworthy bodies”, “culturally disadvantaged bodies”, and “bodies at risk of political misuse and emotional suffering”. The themes are further described below beginning with the structural factors and then the specific descriptors that youth use to refer to their bodies.

### 3.2. Economic and Living Standards Factors

Although young people were initially asked to give a description of health and wellbeing of their communities, there were common economic descriptors adopted by all youth from the different regions in Kenya. Access to meaningful employment, quality education, housing and working/employment conditions, cost of living, and economic inequality were considered important descriptors by all the participants. Economic opportunities were related to having income that allows youth to have access to basic needs, such as food and good housing. One participant in a female only FGD in Kibera, Nairobi County described a healthy community and having a good life as:

“You know when you have money, you will buy for your family the food that they want and that will make them healthy and the community at large”. In Makueni County, similar sentiments were echoed where a participant in a male FGD described a healthy community as one where “the people are able to raise their children well by providing them shelter, education, and also food”.

(female youth in Makueni County)

In Nyanza and Makueni, regions of relatively low socioeconomic status and unfavorable climatic conditions, many participants considered their communities to be unhealthy and felt that their communities would only be considered healthy if income and employment challenges were addressed:

“If we have a source of income that that will make us have a good life and healthy community…job availability can bring a good life. I talk of employment, if you got employment, employment would make our life, or our health become better. This one will result into a healthy community”.

(female youth in Kisumu County)

The youth in these regions also associated the lack of employment to the regions’ political inclination and the ethnocentric governance that have tainted Kenya’s history. Since Kenya’s independence, these regions have consistently remained in opposition government and the young people consider themselves disadvantaged in terms of employment access, infrastructural development for schools, business, and healthcare facilities.

With reference to sociocultural and regional variations, the male youth were more concerned about the unemployment rate than the female youth. In the arid to semi-arid and the socioeconomically disadvantaged regions, wellbeing was defined in relation to income, inflation, and wealth poverty. A plausible explanation for this observation is that in societies experiencing high unemployment and where household provisioning is seen as a sole responsibility for the men, the males, including the youth, are concerned about the issues of unemployment. Specifically, when there is a mismatch between education and employment, people’s conceptualization of a healthy community and a good life is negatively affected. The youth narrated how some of them have college or university certificates but are frustrated as their aspirations do not match their achievements. To cope with the associated distress, some resort to social behaviors such as crime, alcoholism, and substance abuse.

Conversely, the youth in Nyandarua County perceived the health and wellbeing of their community to be good, especially in relation to the availability of opportunities. Notably, at the time of this study, most of the youth in this region were engaged by the government in the National Youth Service. Though temporary, this employment opportunity impacted positively on how the youth perceived the health and wellbeing of their communities. For instance, a female indicated that “now I have a job. So, I can say this community is a healthy community”.

### 3.3. State of the Environment and Neighborhood Effect

In general, the findings reveal that aspects of the surrounding environment—personal safety, security, cleanliness, climate change, food and water insecurity, status of the infrastructure, and the emergence of ghetto communities around the gated settlements—inform people’s perceptions of health and wellbeing. In the formal settlement for instance, the people were more concerned about the mushrooming informal settlements that have engulfed their societies, creating a sense of insecurity and filthy neighborhoods. Insecurity in these contexts could either be an actual or perceived increase in crime rates that may be driven by the difference in the social class between those in the formal and informal settlements. Specific to the youth in the formal settlements, the informal settlements could be areas for black markets for illegal drugs targeting the youths.

Water and food scarcity were mentioned as important to health and wellbeing in all the regions, but more frequently in the rural communities than in urban areas as earlier stated. Even though less frequently used as a descriptor of healthy community and wellbeing, water scarcity and quality and changes in the climatic conditions were a concern to the female participants. This could be because women are bestowed with the responsibility of ensuring that their families are water secure. At the time of data collection, Kenya was experiencing a massive drought that struck the horn of Africa and the eastern side of the continent. Water and food are basic commodities and their inaccessibility, quality, affordability, safety, and time spent looking for these commodities were found to have an influence on people’s perceptions of a healthy and a flourishing life. In Makueni County, the youth considered their community to be unhealthy:

“I can say that the community we are living in is not healthy, the health status is not good because the environment is not conducive…most of the people from this area fetch water from the river…and water…is polluted. It is not clean. This one leads to many people suffering from different diseases, such as bilharzia, those waterborne diseases. Also, there is unreliable rainfall throughout the year…people experience famine and drought…the cattle or the livestock are not surviving they are dying…there is low production of food…so, shortage of food also leads to malnutrition of the youths, the young children, and the aged, so that one makes life too difficult and the community unhealthy”.

(FGD—male youth in Makueni County)

### 3.4. Social Relationships and Interactions

The findings reveal that according to young people, a healthy community is one in which people live in solidarity as it relates to the willingness of members of a society to cooperate with each other for the purposes of survival and contribution to a wide variety of outcomes such as health and economic prosperity. The feelings and experiences of social capital and social inclusion, having social networks, and the ability to move into a higher social class were highlighted as important factors to young people. In communities where people felt included in decision-making and community work and supported both socially and financially, participants perceived such communities as healthy and to have a good life.

In Nyandarua County, the existence of youth groups and other social networks has created opportunities for youngsters to access financial aid through loans, hence they are able to start up small businesses to boost their economic wellbeing:

“In this community, we have youth groups where we contribute money together. Through this, we have access to loans of up to KSh. 100,000 (≈USD 1,000). When we get this money, they use it to buy land which is subdivided amongst the group members or this money is used in different developments. This is helpful to us as young people”.

(FGD—female youth in Nyandarua County)

Similar sentiments were also echoed in Kibera, an informal settlement in Nairobi County, where young people come together for cleanup activities to ensure that neighborhoods have some level of hygiene and sanitation.

In contrast, social cohesion emerged as an aspiration desired by youth in Kisumu County and the formal settlement in Nairobi, with many respondents concerned about the loss of social connection. Social trust among individuals was perceived to have diminished as people become more individualistic and are less concerned about their neighbors. This is what a female youth in Kisumu county said:

“For our community to be healthy, we need to have a component of love. We need to love one another…so that in case one of us is unable to send their child to school, the community can come together…to help this child get education. By doing so, we will find that such children can come back and help her society”.

(FGD—female youth in Kisumu County)

### 3.5. Community Health Status and Girls as “Bleeding Bodies”

The quality of healthcare services as well as individual physical and emotional health were major descriptors of personal and societal health and wellbeing. A healthy community was considered by the participants as one that is free from disease and stress and one where the healthcare needs of young people are met. Some healthcare challenges included drug availability in health facilities and access to condoms. The need for condoms contributes to the spread of sexually transmitted diseases, including HIV, among youth in all socioeconomic areas and is a major health issue affecting young people:

“STIs, maybe gonorrhea, this comes from the point of pleasures for young people…We are not accessing condoms; we end up getting infected with STIs”.

(FGD—male youth in Nyandarua County)

An aspect of community health status that emerged as a critical factor was menstrual hygiene. As female youth undergo biological changes in a resource-constrained environment, they experience traumatized menstrual periods that occur in the absence of sanitary towels and basic hygiene services. The female youth construct their health and wellbeing around issues of menstrual hygiene and consider themselves as “bleeding bodies” at risk of soiling their clothes and falling victim to early pregnancies. Most participants identified fear and isolation during menstruation and the resulting physical and mental health effects as some of the barriers to enjoyment of life:

“Wanting to enjoy life responsibly and fear of the monthly period. Provision of sanitary towels is something that…is a challenge to the girls in this community, especially when they start to get such changes, we start to have certain fears”.

(ID—female youth in Kisumu County)

Female youth experience moments of embarrassment and loneliness around menstruation and experience challenges in accessing hygiene facilities (e.g., toilets or latrines), clean water for bathing, soap, underpants, and sanitary pads. They adopt coping strategies, such as using old clothes for sanitary towels, which they wash and reuse. However, this negatively impacts their physical health because of the scarcity of clean water and a lack of soap, which increases the risk of infection and disease. The lack of access to sanitary pads also negatively impacts their mental health, being in fear of soiling their clothing.

In circumstances of hardship, menstruation can lead to other social issues. There is an erroneous belief that contraceptives will stop menstruation, for example, and transactional sex may be exchanged for sanitary pads, underpants, and financial support. One participant, a 17-year-old orphan who had dropped out of school due to pregnancy, narrated her experience with menstruation and early marriage:

“For me, it has brought me a serious challenge…when I asked my grandmother for this stuff, she was always saying that she had no money to buy such kind of things. So, I had to look for money to get sanitary towels…I was forced to get a boyfriend. He used to buy me pads and that’s how I ended up conceiving [deep breath] and marriage [was] the only option”.

(IDI—female youth in Kisumu County)

These observations were common in regions of low socioeconomic status where basic needs—shelter, food, and clothing—were lacking. In these settings, sanitary pads were considered a luxury, and caregivers were more concerned with providing basic needs. In such instances, social networks were identified to be a vital source of support to young women during menstruation. However, such systems were unstable and would disintegrate quickly if people became more concerned about their immediate families.

### 3.6. Parental Guidance Gap and “Untrustworthy Bodies”

The discussion from both male and female youth in all the study sites indicated lack of proper parental guidance as a source of some of the challenges they experience as it related to health and wellbeing. The young people are concerned about parental neglect in a busy neoliberal world which is contributing to lower levels of social, economic, and emotional wellbeing. As parents get busy and spend less time at home with their children, whose lives are now dependent on the domestic house workers, the youth are unable to share their lives with them; instead they resort to alternative sources of happiness, but still suffer both emotionally and physically.

“…the parent does not have time for the youth, for that matter. And then most of them die from inside, they just keep quiet, they think it will heal over time”.

(IDI—male youth in Langata)

Lack of parental engagement and leadership not only cause physical health challenges, but also emotional suffering, which is associated with the rise in suicides and low life expectancy for youth:

“People are committing suicides every now and then. Find that a young man has committed suicide because of a lady he wanted to marry…Some of them, they commit suicide because of the issues that emanate from the house or just because of relationships”.

(FGD—female youth in Kisumu County)

The female youth felt their parents lacked trust in their ability to take care of themselves. For this reason, they felt a lack of freedom to interact with other males and females in the community, thus limiting their personal autonomy and personal development and negatively impacting their perceived wellbeing:

“You will find that we are denied freedom of interaction…you are a girl, so your life is that of being held in the house. You are taken to school and brought back home, and you are enclosed indoors”.

(FGD—female youth in Langata)

Socioeconomic inequalities, political violence, and unemployment were identified as factors that created avenues for untrustworthiness. These inequalities impacted negatively on the individual self-image of male youths as associated with political violence and crime. From all the study sites, the male youths felt that their communities did not trust their ability to be productive members of society, and their initiatives were often viewed with suspicion. They reported that income generated from activities such as chicken rearing, crop farming, or artwork were often imagined to be from criminal activities, thus creating barriers to market opportunities:

“Lack of trust within the community. You will find that if you rear chicken and you want to sell it in the market, people will ask you, where have you gotten this chicken? It is like people believe that us as youth, we steal from other places. So, getting a market for our produce…a challenge in this community. This affects us”.

(FGD—male youth in Nyandarua County)

### 3.7. Culturally Disadvantaged Bodies

It was found that participants socially construct their health and wellbeing in relation to social and psychosocial health impacts associated with perceived disadvantages that emerge from living in a neoliberal society characterized by the culture materialism and consumerism. Societal expectations and relative comparisons exert psychosocial stress on youth. A representative from the community-based organization working with youth reported that:

“For the young adults and the teenagers, they’re people who want to look like others. They want to be like celebrities, so they tend to behave, dress like them…I can say the lifestyle here is more of experimenting because people are not taught about self-awareness and this exerts social pressure on the youth”.

(CBO representative who was a youth working with youths in Kibera)

Despite the numerous gender equality interventions that aim to enhance their capabilities, females in poor areas were observed to be disproportionately disadvantaged compared to males. This could be because of lack of contextualization of such policies which are more informed by the global development agenda. Participants observed that females are constitutionally but not traditionally allowed to own land, a condition they felt contributed to a lack of material capital in female-headed households:

“Like now in my community, if I give birth to a child out of wedlock and the man damps me and, in my home, I am not given land, this brings what I would call multiple problems…I will not be able to start off my life again and even do some farming to take care of my children…it is a real challenge to us as girls”.

(FGD—female youth in Langata)

While female-headed households are not traditionally acceptable, the practice of early marriage is still common with higher rates in arid and semi-arid regions, as well as areas with high poverty rates. Early marriage disproportionately affects female youth and contributes to gender inequality in many avenues of growth, including access to education. Notably, however, some male participants felt that gender equality efforts focus on females and they felt neglected and unable to meet societal demands. Society expects the boys to demonstrate their manliness by starting to provide, but if women and girls are prioritized, they are unable to meet this obligation. A need for further attention to males emerged in interviews and was emphasized by male participants as a programmatic priority for community-based and non-governmental organizations.

### 3.8. Bodies at Risk of Political Misuse and Emotional Suffering

Participants called for good leadership and governance that propagates real engagement of young people in decision-making and noted that the lack of employment makes them vulnerable during political campaign seasons, as their electoral rights are violated through bribery. Also of importance is the lack of coercion due to tribal politics which has created cycles of political uncertainties. This affects male youth in particular and could potentially result in reduced participation in political decision-making for both male and female youth. A 23-year-old girl narrated her experience with politics and how she has lost interest in voting:

“I used to engage in politics sometimes, but I want to testify that I left it. There is a time we went for campaign for one of the candidates and chaos erupted. On that day, I just escaped death narrowly. From that time, I said never, never again to engage in matters of politics”.

(FGD—female youth in Kisumu County)

Electoral violence, voter bribery, and manipulation limit collective and individual agency, hence compromising the democratic rights of the participants, which were highlighted as perceived determinants of a healthy community. The implication is, therefore, that there is a lack of integrity and that political ideologies or manifestos do not resolve social and political challenges facing the community as politics in the country is driven by ethnicity rather than political beliefs.

“The politicians, they take advantage over us because we are poor and have nothing. We sell our votes for about 3000/– ($30). So, our votes do not have the power to vote out a bad leader because you have already sold your vote”.

(FGD—male youth in Kibera)

As previously mentioned, Kenya’s politics has been characterized by ethnolinguistic identity, involving the creation of and voting for political parties along tribal lines rather than political ideologies and policies [27]. Such biases are founded on the belief that political parties offer ethnic groups the only hope to rise to power and enable their communities to share state resources with their members [25]. To this end, participants considered the trajectory of political structures since independence to be critical to achieving a flourishing life. For instance, participants passionately criticized ethnicity and ethnic politics which favored a section of the population, especially with reference to resource distribution. The participants further observed the perceived effects of ethnicity on governance and its implications to health and wellbeing.

## 4. Discussion

To the best of our knowledge, this is the first paper describing meaning and perceptions of health and wellbeing as described by youth from different settings—urban, rural, and the arid to semi-arid regions of Kenya. Young people’s perception of health and wellbeing are not uniform and show a wide variation between the male and females as well as regionally. In this study, young people demonstrated that interactions with place and the existing systemic factors are important descriptors of concepts of health and wellbeing [47]. The findings reveal that the availability of economic opportunities, social interactions, and neighborhood effects are important descriptors of health and wellbeing. These findings support the results of previous studies that highlight the role of socioeconomic characteristics of place such as the availability of social capital and cohesion, income, employment opportunities, and housing and food security in influencing perceptions and meanings that people attach to their health and wellbeing [48,49]. In relation to lived experiences, the findings reveal that youth in Kenya socially construct their health and wellbeing around specific issues such as menstrual hygiene, trust, culture, and perceived risks. While the overall state of health and quality of life as it relates to culture, trustworthiness, and risks to emotional and physical health are top priority for all youth, there are specific concerns along gender lines.

Female participants socially constructed their immediate and long-term health and wellbeing around menstrual hygiene issues. The findings show that girls transitioning into womanhood within resource-constrained areas characterized by inequality and inaccessibility of basic hygiene services (including access of sanitary pads) experience feelings of embarrassment, loneliness, fear, and trauma associated with menstruation, as they are apprehensive of early pregnancy and perceived themselves as bleeding bodies at risk of soiling themselves. Research has shown that menstruation affects structural aspects of the school environment, including social and physical settings, school attendance, and academic success [50]. Furthermore, survival strategies such as the use of cloths when pads are not available or not affordable can pose health challenges in areas that lack access to clean water, soap, or toilets [51,52], and the need for these supplies and money can lead to transactional sex, which may have psychosocial and physical health effects, including the loss of self-esteem, unwanted pregnancies, and sexually transmitted diseases.

Another finding in our research was a feeling of lack of personal autonomy brought on by strict parents who did not trust their children to safely interact with others and not engage in risky sexual behaviors. This particular finding is a function of the perceived risk of early pregnancy, which is a common occurrence in contexts characterized by social and economic inequalities [53]. In these contexts, parents tend to overprotect their children and monitor female youth. It is an experience that the participants associated with reduced personal autonomy and self-sufficiency and a limitation of their own agency. Previous studies have shown both advantages and disadvantages of parental strictness on youth. Parental control may have positive impacts, such as adequate sleep and good health outcomes, if youth are not allowed out alone at night and thus do not participate in high-risk behaviors, such as attendance of night discos and dance clubs [54,55]. However, over-vigilance by the parents may contribute to psychosocial health problems, especially increased depressive symptoms [56]. The findings of our research confirm this as female youth, especially those from middle-class households, suggested that a sense of independence would be important in achieving a level of personal agency and promoting and sustaining a high quality of life.

Male participants were often concerned about a lack of trust and being associated with criminal acts. The lack of community trust limits economic opportunities and the social and emotional wellbeing of these individuals. Social, economic, and political inequalities, especially in societies with high rates of unemployment, contribute to this perception [57,58]. In Kenya, men are socialized as family heads and sole providers. They are also influenced by capitalist society and its culture of materialism, self-interest, and consumerism. Media perpetuates the idea that happiness and a good life is likened with what you own or consume, which is often beyond the reach of many youth [59]. When this culture of consumerism interacts with societal norms, beliefs, and traditions that promote inequality and place pressure on male youth to prove their manhood through income and consumer goods, health and wellbeing is negatively affected, including increased levels of anxiety and stress due to societal expectations. This is particularly true when opportunities for income generation are unavailable. Evidence shows that some people may then resort to criminal activities to achieve status as a provider and leader. However, increased criminal activity creates a lack of trust of male youth in their communities [58,60], with all male youths appearing suspect and are associated with criminal activities.

Of critical importance to both the male and female youth are the effects of electoral violence and the executive’s perceived autocratic leadership. Voter bribery compromises human agency and the efforts of activist groups toward empowering the electorate toward informed choice of leadership. In such circumstances, real engagement in political decision making by the citizenry is equally not guaranteed, hence, their democratic rights are violated [61]. Additionally, the unintended consequences of politics such as political unrest and riots also affect people’s active participation in politics. In setting global and national development agendas that aim to lay foundations for discourses to improve quality of life and health for all Kenyans, the issues highlighted in this paper are important if the voices of youth are to be reflected in the national and local development agenda.

We recognize the limitations of this study, which was a qualitative study across several regions, but the results cannot be generalized to the whole Kenyan population or other LMICs. Although a there are several shared experiences among adolescents, it was beyond the capacity of this study to collect data on the trend and frequency of occurrence of the specific issues mentioned and the conclusions drawn here are based on the FGD and IDI data. The data was also not disaggregated by age and hence may be limited in this aspect. Finally, the study relied on self-reported data, which is prone to social bias, especially with respect to personalized experiences of menstruation and sexual behavior.

## 5. Conclusions

Understanding the aspects of health and wellbeing that matter most to the youth is a critical step toward the creation of a localized development agenda. This is because young adults are not a homogenous group as their concerns about health and wellbeing are diverse and vary by region, social class, ethnicity, and gender. We suggest that an understanding of situated discourses—including the socioeconomic characteristics of place—such as availability of income-generating activities, social cohesion, neighborhood characteristics, and historical and political discourses are essential in the development of policies and interventions that aim to improve the quality of life and wellbeing for young adults. It is against this backdrop that our study demonstrates that female youth in poor communities experience feelings of embarrassment, loneliness, fear, and trauma, as they perceive themselves at risk for early pregnancy and as bleeding bodies at risk of soiling themselves. Additionally, girls experience a lack of personal autonomy due to the over-vigilance of parents who see female bodies as delicate and at risk of early pregnancy. Male youth, however, are viewed as untrustworthy and are often associated with criminal acts and violence. With limited employment opportunities and the experience of lack of trust, male adolescent Kenyans are frustrated, thus depicting poor economic and psychosocial health and wellbeing outcomes. As the world works toward securing healthy, peaceful, and prosperous life for all, understanding these pertinent areas of health and wellbeing that matter to young people is important if we are to alleviate human suffering today and into the future.

## Figures and Tables

**Table 1:** Focus group discussion (FGD) and in-depth interview (IDI) sample characteristics.

Data Source	Characteristics	Region/County	Kisumu	Makueni	Nyandarua	Nairobi-Langata ^†^	Nairobi-Kibera ^‡^
**FGD participants**	**Age (range)**	15–24	20 (17–24)	17 (16–19)	17 (18–24)	11 (17–24)	21 (18–24)
		No. of FGDs	2	2	2	2	2
	**Gender**	Male	12	8	9	4	11
		Female	8	9	8	8	10
	**Education level**	Primary and below	13	12	2	2	8
		Secondary and above	7	5	15	9	13
	**Household size**	Range	1–15	2–13	1–10	1–8	1–12
	**Range of monthly income (in USD)**		0–300	0–250	0–325	0–900	0–275
**IDI participants**		No. of IDIs	4	2	2	3	3
	**Gender**	Male	1	1	1	2	2
		Female	3	1	1	1	1
	**Household size**	Range	2–13	2–10	2–5	2–8	2–5
	**Education level**	Primary and below	2	0	0	0	0
		Secondary and above	2	2	2	1	2
	**Range of monthly income (in USD)**		5–100	50–100	10–100	5–500	5–200

^†^ Formal settlement in Nairobi Province (Langata gated communities); ^‡^ informal settlement in Nairobi Province (Kibera).
